# Effect of external subretinal fluid drainage on persistent subretinal fluid after scleral buckle surgery in macula-involving rhegmatogenous retinal detachment

**DOI:** 10.1038/s41598-023-49719-5

**Published:** 2023-12-13

**Authors:** Jae Rok Do, Dong Ho Park, Jae Pil Shin, Yong Koo Kang

**Affiliations:** 1 Department of Ophthalmology, School of Medicine, Kyungpook National University, Daegu, Korea; 2 Department of Ophthalmology, Nune Eye Hospital, Daegu, Korea

**Keywords:** Eye diseases, Retinal diseases, Vitreous detachment

## Abstract

This study aimed to analyze the duration and causes of persistent subretinal fluid (PSF) after scleral buckle (SB) surgery in patients with macula-involving rhegmatogenous retinal detachment (RRD). Sixty-one eyes of 61 patients with macula-involving RRD who underwent SB surgery between 2016 and 2022 were reviewed retrospectively. PSF was confirmed on optical coherence tomography. The PSF duration after surgery and the analysis of relevant ocular and systemic factors were conducted according to the PSF duration. The mean duration of PSF was 5.9 ± 4.6 months in all eyes and 8.1 ± 5.0 months in eyes not treated with external subretinal fluid (SRF) drainage, which was significantly longer than 4.5 ± 3.7 months in those subjected to external SRF drainage. The mean best-corrected visual acuity improved significantly 3 months after surgery. There were significant visual improvements in the external SRF drainage group compared to the non-drainage group during all follow-up periods. Longstanding shallow RRD was significantly associated with longer PSF duration after SB surgery. External SRF drainage during SB surgery can effectively reduce SRF, shorten the duration of PSF, and accelerate visual improvement.

## Introduction

Great technological advance in vitreoretinal surgery and the introduction of small-gauge pars plana vitrectomy (PPV), there was increasing trend toward PPV for RRD repair surgery^1^. However, scleral buckling (SB) has clear advantages over PPV and is still remained as a primary treatment option of RRD in younger, phakic, proliferative vitreoretinopathy, and high myopia cases^2,3^.

Persistent subretinal fluid (PSF) is defined as the continued presence of subretinal fluid (SRF) between the sensory retina and the retinal pigment epithelium (RPE) seen on optical coherent tomography (OCT), despite successful reattachment of the retina and sealing of all retinal breaks^4,5^. The incidence of PSF varies widely according to differences in baseline patient demographics, characteristics, and surgical procedures^4–8^. Previous studies have also reported that younger patients and those with macula-off RRD have a higher incidence of postoperative SRF and delayed absorption^9^. PSF may cause metamorphopsia and the loss of depth perception^10^. However, whether PSF influences visual prognosis remains unclear^11–13^.

The SRF drainage procedure is especially useful when excessive fluid impedes the attainment of adequate buckle indentation required to close the break. However, it is one of the most critical steps during SB surgery because of the risk of complications like vitreous incarceration, retinal perforation, subretinal hemorrhage, and choroidal detachment^14,15^. Nevertheless, the drainage procedure is necessary to facilitate precise localization of retinal breaks, allow the attachment of the retina within the area indented by the buckle, and reduce intraocular pressure, allowing more effective scleral indentation.

The purpose of this study was to investigate the incidence and associated factors of PSF following SB surgery for RRD repair. In addition, we also assessed the effect of external SRF drainage during SB surgery on the duration of PSF.

## Methods

Medical records were retrospectively reviewed after approval of the Institutional Review Board of Kyungpook National University Hospital (IRB No. 2023-06-020), and the requirement for informed consent was waived because of the retrospective nature of the study by the Institutional Review Board of Kyungpook National University Hospital. The review was conducted in accordance with the tenets of the Declaration of Helsinki.

### Study participants

We included patients diagnosed with macula-involving RRD who underwent segmental SB surgery alone and achieved successful retinal reattachment between January 2016 and September 2022.

The inclusion criteria was as follow: (1) eyes with macula-involving RRD; (2) eyes with SRF on preoperative macular OCT; (3) eyes with persistent SRF on postoperative OCT scans; (4) eyes achieved reattachment of the retina and sealing of all retinal breaks after surgery and completed at least 6 months of follow-up with OCT examinations. If the patient had macula-involving RRD in both eyes, we only included the more severe eye.

Patients were excluded if they met any of the following criteria: (1) history of intraocular surgery; (2) pre-existing retinal vascular diseases such as diabetic retinopathy and vascular occlusive disease; (3) pre-existing macular pathological features such as macular edema, vitreomacular traction syndrome, and epiretinal membrane; (4) history of uveitis or intraocular inflammatory disease; (5) RRD without subretinal fluid on preoperative macular OCT scans; (6) underwent encircling buckle surgery due to severe proliferative vitreoretinopathy; (7) underwent procedures such as pneumatic retinopathy that affect subretinal fluid absorption; (8) used drugs such as acetazolamide that affect subretinal fluid absorption; (9) postoperative complications such as choroidal detachment that affect affect subretinal fluid absorption.

Ophthalmic examinations, including initial best-corrected visual acuity (BCVA) using a Snellen chart, intraocular pressure (IOP) measurement, slit lamp examination, fundus examination, and OCT examination, were performed. Axial length values were measured and the degree of myopia was confirmed in all patients before SB surgery using IOLMaster 500 & 700 (Carl Zeiss Meditec, Jena, Germany).

All examinations were repeated at every follow-up visit after SB surgery. The Snellen chart-based BCVA values were converted to their logMAR equivalents (logarithm of the minimum angle of resolution) for statistical analyses. The presence of macula-involving RRD was confirmed by a preoperative OCT scan of the macula. The volume mode scan of a 6 × 6 mm area was performed using the spectral-domain OCT (Spectralis®, Heidelberg Engineering, Heidelberg, Germany). The presence of SRF was defined as a clear space between the photoreceptor layer and the RPE on OCT. PSF was defined as SRF present within any area on 6 × 6 mm OCT scans that persists after SB surgery. Using the built-in ruler in the software program, the subfoveal choroidal thickness (SFCT) and subretinal fluid height (SRFH) were manually measured. The SFCT was measured by determining the vertical distance from the outer surface of the foveal pigment epithelium to the inner surface of the sclera. Meanwhile, SRFH was measured from the outer interface of the neuroepithelial layer to the inner surface of the pigment epithelium^16^.

### The surgical technique

All SB surgeries were performed by two experienced surgeons (DHP and YKK). All surgeries were digitally recorded as per the standard procedure. In all patients, the standard technique of SB retinal detachment surgery was followed. The area of the segmental buckle was decided based on the size, number, and location of the retinal breaks. An indirect ophthalmoscopic evaluation was performed intraoperatively to identify and localize all retinal breaks.

The SRF drainage procedure was conducted in cases with large amounts of subretinal fluid that unable to make adequate position of the buckle. The SRF drainage site was determined based on the extent of retinal detachment and the position of the retinal breaks (preferably distant from the vortex veins of the choroid).

The drainage procedure was performed through a radial scleral incision after diathermy to reduce the risk of bleeding. Choroidotomy was carried out using external laser choroidotomy or needle draiage technique. For needle drainage, a choroidotomy was created a 27-gauge needle to reach the subretinal space. For laser choroidotomy, the endolaser probe was held in close proximity and perpendicular to the choroidal surface. It was used to create the choroidotomy, ensuring the prevention of any retinal damage^17^. A progressive scleral depression was maintained to maximize SRF drainage while avoiding excessive eyeball pressure and depression. The procedure was continued until a cluster of pigments appeared in the drained SRF and was terminated after the pigment disappeared^18^. Indirect ophthalmoscopy was conducted intraoperatively to confirm SRF drainage at the end of the procedure.

### Statistical analyses

Statistical analyses were performed using the SPSS Statistics software version 20 (IBM Corp., Armonk, NY, USA). A Kaplan–Meier graph was plotted to depict the survival time of PSF and the log-rank test was used to compare the mean duration of PSF. Repeated measures analysis of variance corrected by the Bonferroni method was used to compare the mean BCVA according to the follow-up periods. Mann–Whitney U test was performed to evaluate the duration of PSF according to high myopia of axial length, and the visual improvement from baseline to the follow-up periods based on the procedure of external SRF drainage. Ocular and surgical factors that affected the duration of PSF were analyzed using univariate and multivariate linear regression analyses. For all statistical tests, a *P-*value < 0.05 was considered significant.

## Results

### Demographics of patients

A total of 145 patients were initially enrolled, but 84 patients were excluded. Thus, a total of 61 patients (61 eyes) were enrolled in this study. The demographic and clinical characteristics of all patients are summarized in Table 1.

**Table 1:** Demographic and clinical characteristics of patients included in the study.

Characteristics		Value
Number of eyes, n (%)	OD	40 (65.6%)
	OS	21 (34.4%)
Sex, n (%)	Male	40 (65.6%)
	Female	21 (34.4%)
Mean age, years		28.7 ± 12.2
Mean follow-up periods, months		21.0 ± 16.6
Axial length, mm		26.18 ± 1.65
High myopia, n (%)		24 (39.3%)
Baseline IOP, mmHg		14.6 ± 3.2
Area of RRD, degrees (clock hours)		168 ± 65 (5.6 ± 2.2)
Presence of initial symptom, n (%)		44 (72.1%)
Feature of RRD, n (%)	Bullous	18 (29.5%)
	Shallow	43 (70.5%)
History of other ocular surgery, n (%)		8 (13.1%)
History of atopy, n (%)		8 (13.1%)
Status of fellow eye, n (%)	No treatment	17 (27.9%)
	Laser retinopexy	39 (63.9%)
	Surgery	5 (8.2%)
Subretinal fluid height, µm		506.4 ± 342.3
Subfoveal choroidal thickness, µm		304.4 ± 55.6
External SRF drainage, n (%)		37 (60.7%)

Values are presented as the mean ± standard deviations.

IOP, intraocular pressure; RRD, rhegmatogenous retinal detachment; SRF, subretinal fluid.

Among the 61 patients, 40 were male, and 21 were female. The mean age of all patients was 28.7 ± 12.2 years, and the mean follow-up duration was 21.0 ± 16.6 months. The mean axial length of all eyes was 26.18 ± 1.65 mm. High myopia is defined as the presence of an axial elogantion over 26.50 mm and 24 eyes (39.3%) exhibited high myopia^19^.

A total of 44 patients (72.1%) presented with symptoms of visual disturbance or floaters. Eighteen eyes (29.5%) showed features of bullous retinal detachment, while 43 eyes (70.5%) demonstrated relatively shallow retinal detachment of a chronic nature upon initial fundus examination. The mean area of retinal detachment was 168 ± 65 degrees (5.6 ± 2.2 clock hours).

There was no patient had undergone cataract surgery and only 8 patients (13.1%) had undergone other ocular surgery before RRD repair; 3 underwent strabismus surgery, while 5 had undergone corneal refractive surgery. Additionally, 8 patients (13.1%) had a history of atopic dermatitis. Furthermore, 39 patients (63.9%) were treated with laser retinopexy in the other eye, and 5 patients (8.2%) had a history of surgical treatment in the other eye before RRD repair surgery.

The mean SRFH was 506.4 ± 342.3 µm and the mean SFCT was 304.4 ± 55.6 µm. A total of 37 (60.7%) eyes received SRF drainage. Twenty-four (39.3%) eyes did not receive SRF drainage.

### The duration of PSF

**Figure 1:**
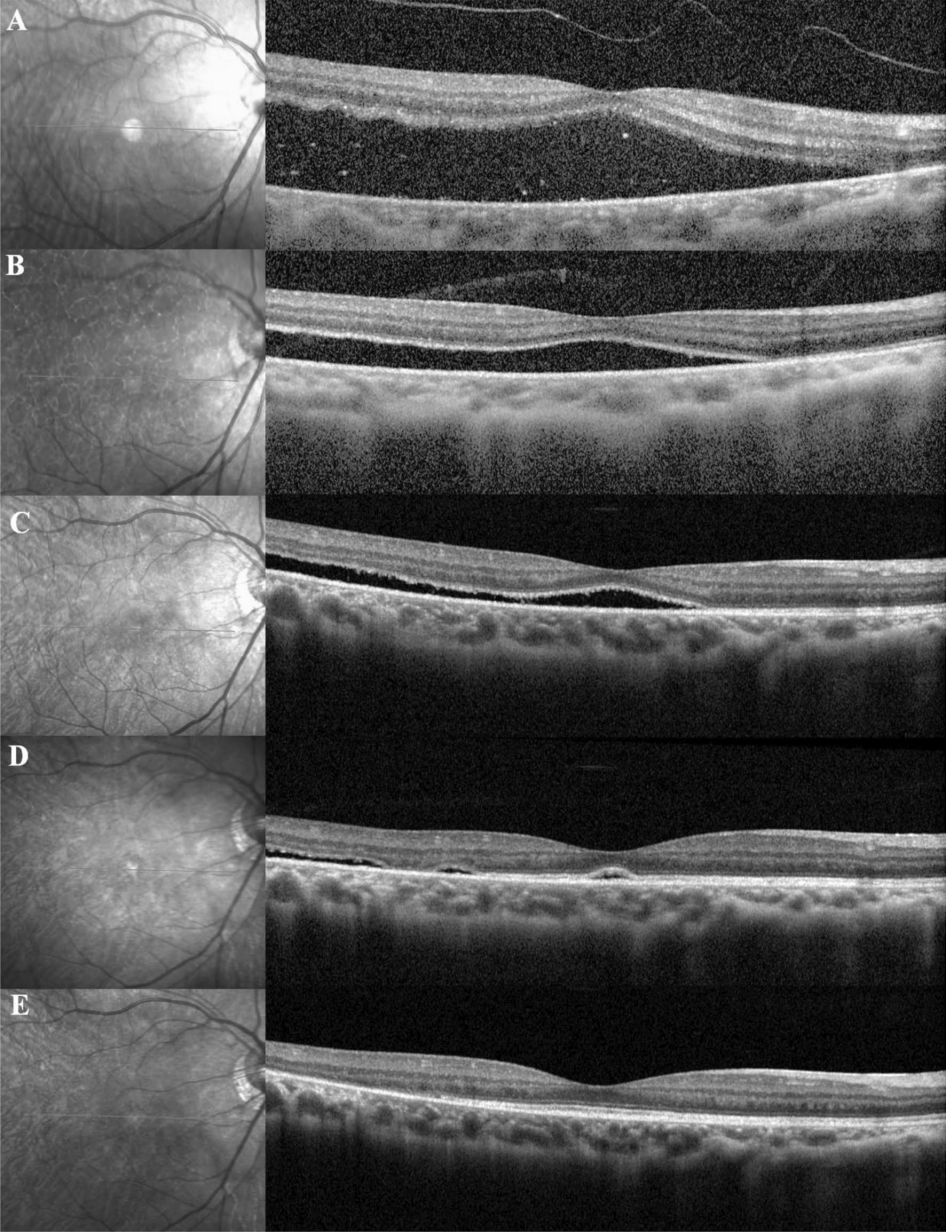
Optical Coherence tomography (OCT) scans of rhegmatogenous retinal detachment with persistant subretinal fluid after scleral buckle surgery in a 27-year-old male. OCT scans at (**A**) Baseline, (**B**) 1 month, (**C)** 3 months, (**D**) 6 months, and (**E**) 9 months after SB surgery with external drainage of subretinal fluid.

The mean duration of PSF was 5.9 ± 4.6 months (range, 0.1–18.0 months) for all patients. On OCT examination, the prevalence of PSF was 83.6% at 1 month, 65.6% at 3 months, 47.5% at 6 months, and 11.5% at 12 months following the RRD repair surgery. Figure 1 shows a representative case of PSF on the OCT scans.

**Figure 2:**
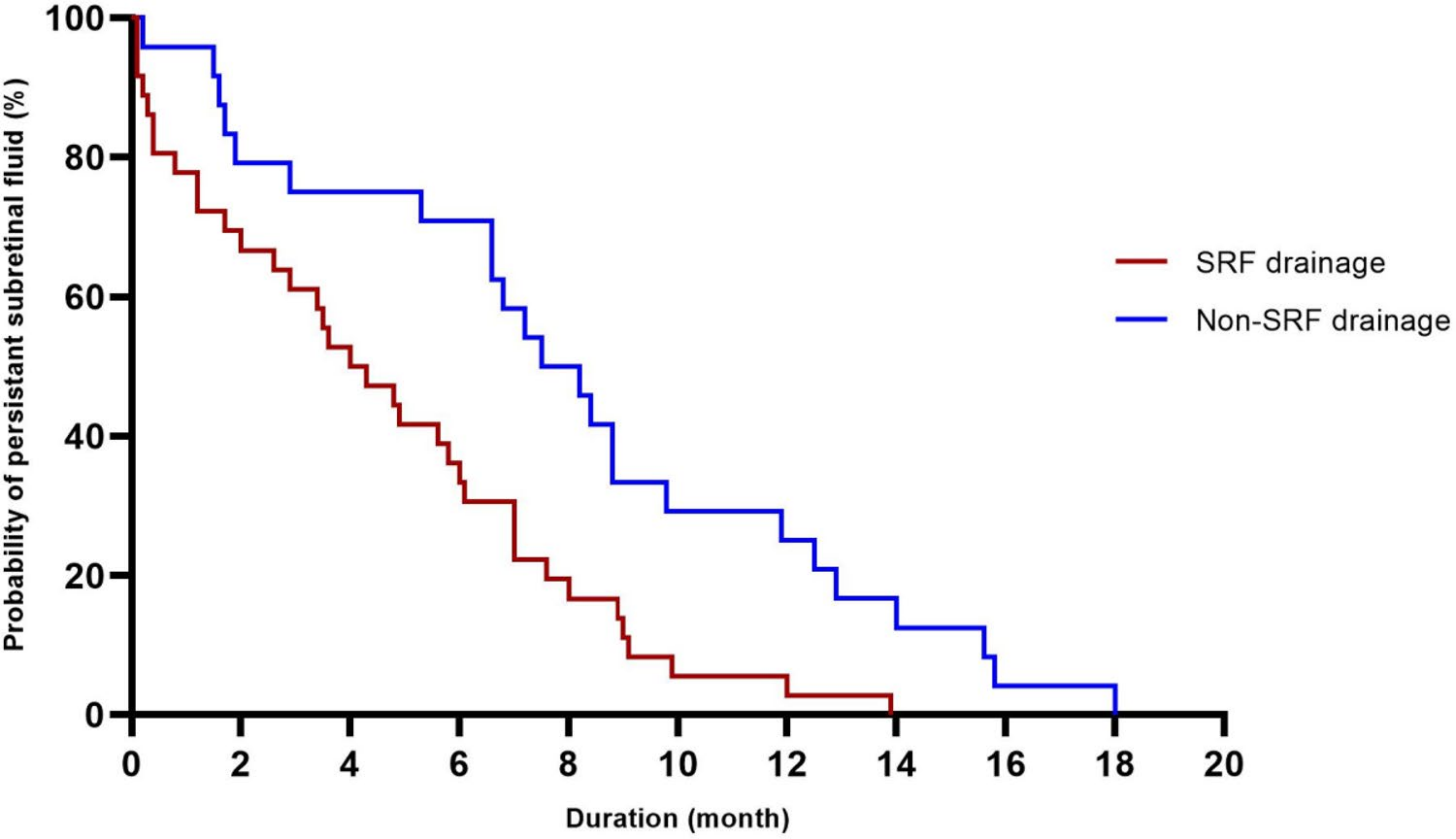
Kaplan–Meier graph illustrating the survival probability of persistent subretinal fluid (PSF) for patients based on whether or not they received external subretinal fluid (SRF) drainage. The mean duration of PSF was significantly longer in eyes without external SRF drainage than in those with external SRF drainage (P = 0.002, log-rank test).

A Kaplan–Meier graph was used to provide an estimate of the survival probability of PSF based on whether or not external SRF drainage was performed, as shown in Fig. 2. The mean duration of PSF in eyes without external SRF drainage was 8.1 ± 5.0 months, which was significantly longer than the 4.5 ± 3.7 months in eyes with external SRF drainage (P = 0.002, log-rank test).

In subgroup analysis according to high myopia over 26.50 mm of axial length, the mean duration of PSF in eyes without external SRF drainage group (n = 7) was 10.4 ± 5.6 months, which was significantly longer than the 4.8 ± 3.6 months in eyes with external SRF drainage group (n = 17) (P = 0.016, Mann–Whitney U test). In non-high myopia group, the mean duration of PSF in eyes without external SRF drainage group (n = 17) was 7.2 ± 4.6 months, which was longer than the 4.2 ± 3.8 months in eyes with external SRF drainage group (n = 20), but not significantly. (P = 0.074, Mann–Whitney U test).

### Best-corrected visual acuity

**Figure 3:**
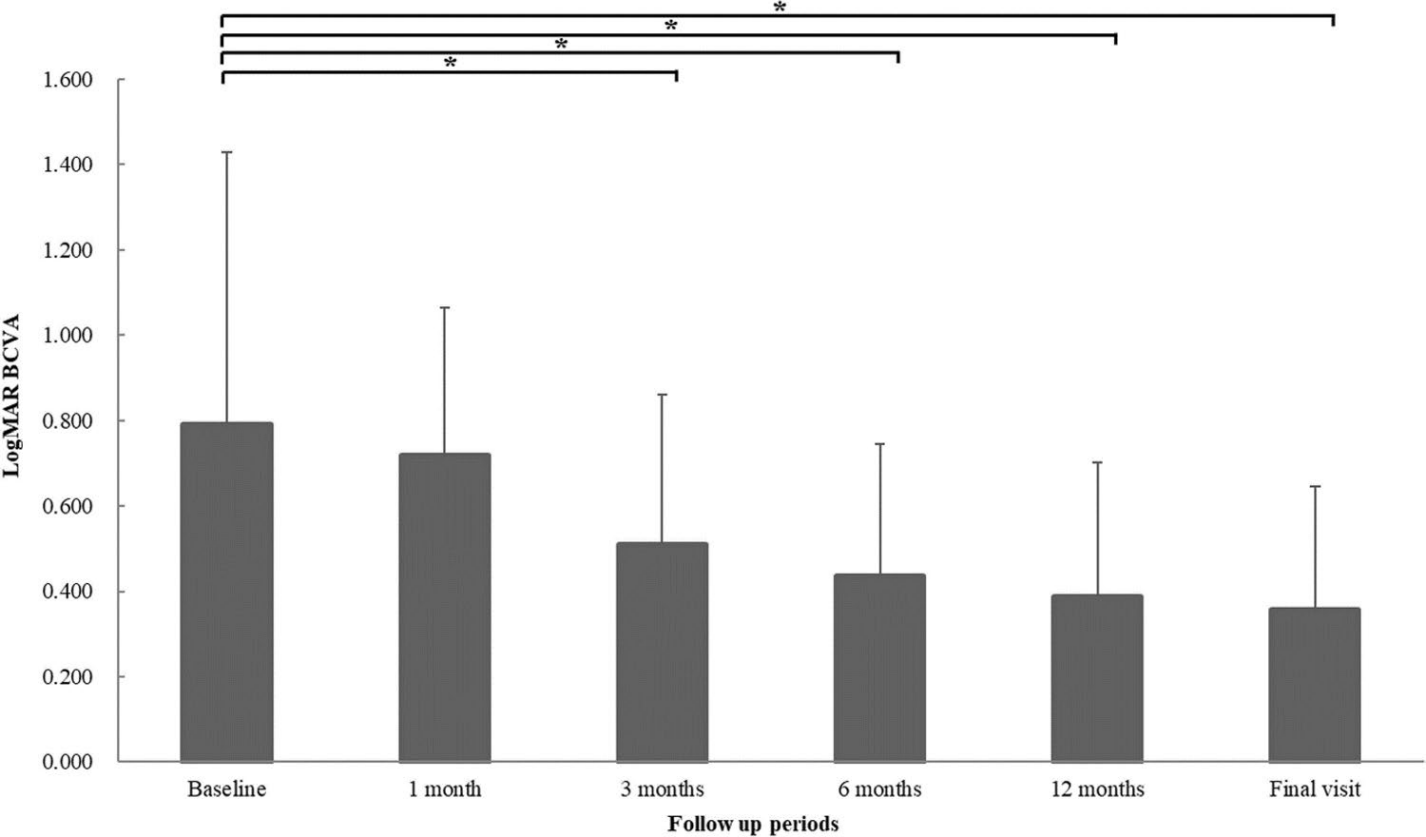
Changes in the mean logarithm of the minimum angle of resolution (logMAR) best-corrected visual acuity (BCVA) during follow-up. The mean BCVA significantly improved three months after surgery compared to baseline (*P* < 0.05, respectively). * *P* < 0.05 by repeated measures analysis of variance corrected by the Bonferroni method.

**Figure 4:**
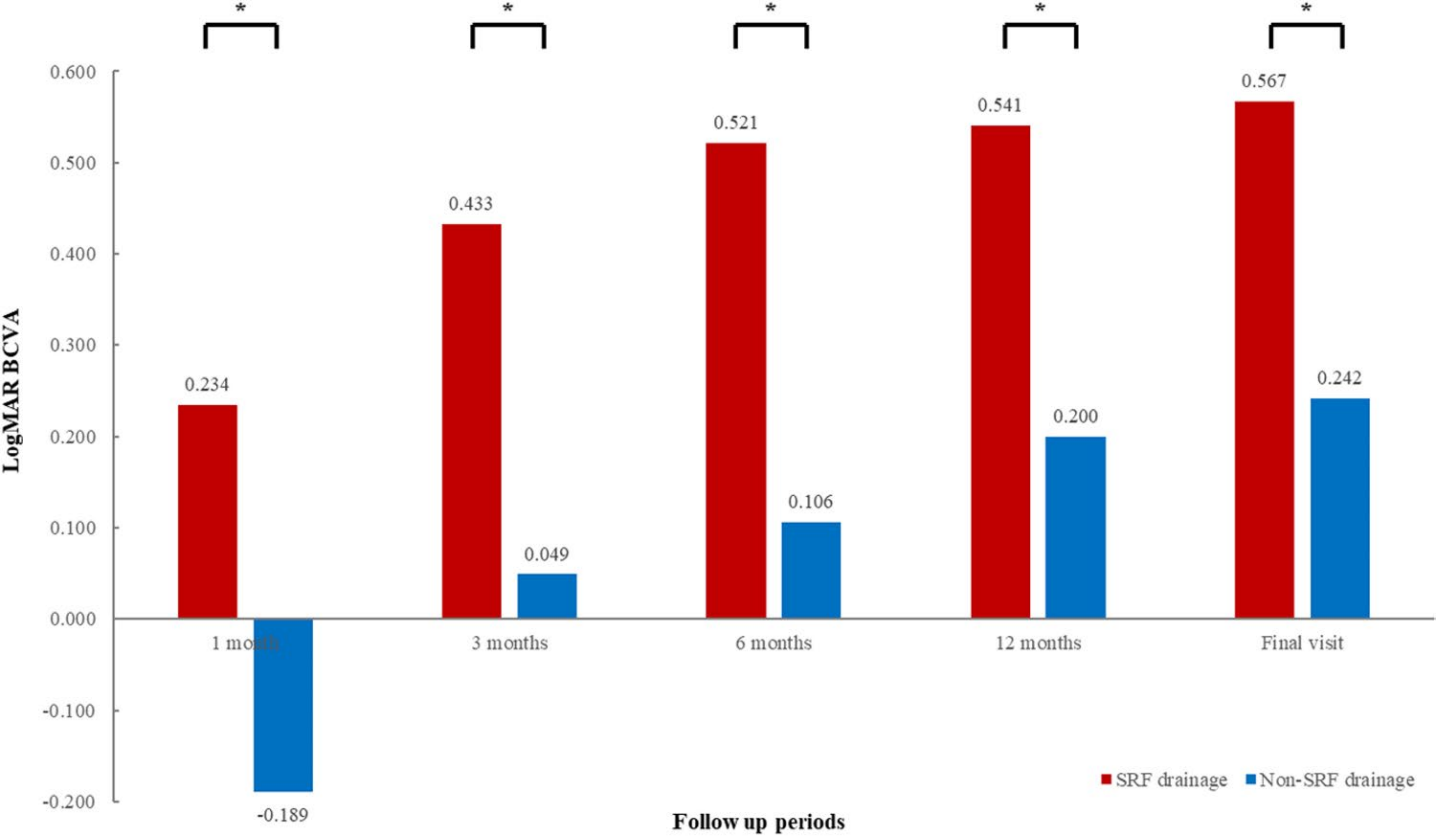
The mean logarithm of the minimum angle of resolution (logMAR) best-corrected visual acuity (BCVA) improvement compared to baseline during follow-up. There were significant BCVA improvements in the external SRF drainage group compared to the non-drainage group during all follow-up periods (P < 0.05, respectively). * *P* < 0.05 by Mann–Whitney U test.

The mean logMAR BCVA of all patients was 0.718 ± 0.634 at baseline, which improved to 0.660 ± 0.454 at 1 month after SB surgery, 0.459 ± 0.456 at 3 months, 0.384 ± 0.440 at 6 months, 0.341 ± 0.429 at 12 months, and 0.313 ± 0.417 at the final visit. There was a statistically significant visual improvement three months after surgery (P < 0.05) (Fig. 3). In the analysis of the external SRF drainage procedure, the mean logMAR BCVA was not significantly different between drainage and non-drainage groups during the follow-up periods. However, there were significant BCVA improvements in the external SRF drainage group compared to the non-drainage group during all follow-up periods: 0.234 ± 0.429 and − 0.189 ± 0.480 at 1 month, 0.433 ± 0.499 and 0.049 ± 0.497 at 3 months, 0.521 ± 0.554 and 0.106 ± 0.490 at 6 months, 0.541 ± 0.583 and 0.200 ± 0.494 at 12 months, 0.567 ± 0.560 and 0.242 ± 0.499 at final visit compared to baseline (P < 0.05, respectively) (Fig. 4).

### Clinical factors associated with the duration of PSF

Table 2 shows the results of multiple linear regression analyses of the associations between clinical factors and the duration of PSF. Non-SRF drainage (*β* = 3.634, *P* = 0.002) and shallow RRD (*β* = 3.197, *P* = 0.013) were significantly associated with the duration of PSF in univariate analysis. In multivariate analysis, Non-SRF drainage (*β* = 3.378, *P* = 0.003) and shallow RRD (*β* = 2.835, *P* = 0.019) were also significantly associated with the duration of PSF (R^2^ = 0.230).

**Table 2:** Univariate and multivariate analyses of associations between clinical factors and the duration of persistent subretinal fluid.

Parameters	*β*	CI (95%)	*P*-value	*β*	CI (95%)	*P*-value
Male sex	0.182	− 2.311 to 2.674	0.885			
Age	0.019	− 0.079 to 0.117	0.695			
Shallow feature of RRD	3.197	0.689 to 5.704	0.013	2.835	0.484 to 5.186	0.019
Area of RRD	− 0.434	− 0.974 to 0.106	0.113			
Axial length	0.153	− 0.570 to 0.877	0.673			
Baseline BCVA	− 0.258	− 2.141 to 1.625	0.785			
Baseline IOP	− 0.268	− 0.637 to 0.102	0.152			
Subfoveal choroidal thickness	− 0.012	− 0.034 to 0.009	0.249			
SRF height	− 0.001	− 0.005 to 0.002	0.414			
Non-SRF drainage	3.634	1.401 to 5.867	0.002	3.378	1.221 to 5.536	0.003
Type of buckling implant	0.519	− 1.805 to 2.842	0.657			

BCVA, best-corrected visual acuity; IOP, intraocular pressure; RRD, rhegmatogenous retinal detachment; SRF, subretinal fluid.

## Discussion

The presence of PSF has been described despite successful RRD repair following both scleral buckling and pars plana vitrectomy^8,20^. It seems to occur frequently; however, the incidence varies widely depending on the reported subjects^7,8,20–22^. Recent studies indicate that patients with younger age, high myopia, or a macula-off status experience a higher incidence of postoperative SRF and delayed absorption^9,23,24^. In addition, Mimouni et al.^25^ reported that intraoperative drainage retinotomy with direct aspiration of SRF decreased the incidence of PSF after vitrectomy.

The need for SRF drainage in SB surgery has been controversial. The subretinal fluid will be absorbed spontaneously if the sclera is adequately indented and the holes are closed^26^. However, the most common cause of failure has been improper buckle position and indentation. In addition, the SRF may shift to the subfoveal area during or after surgery, particularly in patients with macula-involving RRD. For these reasons, several retinal surgeons prefer to perform SRF drainage in a high percentage of SB surgeries, especially in those with severe vitreoretinal traction on the break^14,27,28^.

This study demonstrates that Non-SRF drainage and a shallow RRD feature are significant factors associated with PSF. In other words, patients with longstanding shallow RRD and those who do not conduct external SRF drainage during SB surgery are related to a longer duration of PSF. In patients with longstanding shallow retinal detachment, SRF with high viscosity and oncotic pressure would slow fluid absorption by inhibiting RPE phagocytotic activity^29^. On the other hand, external SRF drainage during SB surgery would reduce the duration of PSF. This procedure not only promotes adequate positioning and indentation of the buckle but also reduces the total quantity of SRF and the amount of SRF that shifts to the subfoveal space. Eventually, this could lead to the rapid absorption of the residual SRF, which is supported by our results.

Previous studies have reported that SRF absorption is related to choroidal blood flow. Kim et al.^24^ reported that eyes demonstrating choroidal vascular hyperpermeability on indocyanine green angiography showed delayed absorption of SRF after RRD surgery. Long et al.^16^ reported that the choriocapillaris flow density in OCT angiography was a factor affecting the absorption of SRF, although SFCT and SRFH were not significantly related to SRF absorption. In this study, thicker SFCT and higher SRFH tended to be associated with longer durations of PSF, but this was not statistically significant.

Fu et al.^23^ recently reported that younger age and high myopia are associated with PSF. They suggested that PSF in younger patients may be related to the composition of the SRF itself because the vitreous is usually less liquefied and more viscous in younger patients^30^. The absorption of SRF depends on passive diffusion and active pumping by the RPE^31^. Patients with high myopia have a thinner and more degenerated RPE, which may be associated with insufficient fluid removal by impaired RPE pumping. Our study showed an association between prolonged PSF duration and patient characteristics such as younger age and longer axial length, although the results were not statistically significant.

The effect of PSF on visual prognosis remains controversial. Borowicz et al.^10^ reported that PSF did not influence the final visual outcome, although PSF may reduce central vision and cause metamorphopsia. However, other reports suggest that PSF is associated with a worse visual prognosis in macula-off RRD patients^6,22^. The authors showed that eyes subjected to external SRF drainage exhibited a shorter PSF duration with faster visual improvement than eyes without external SRF drainage. Thus, external SRF drainage could shorten the duration of PSF and is hence necessary for rapid improvement of visual acuity.

The present study has some limitations. This was a retrospective study with a relatively small sample size. Unfortunately, the collection of a larger sample size was not possible since the frequency of SB surgery for the repair of RRD has decreased, PSF after SB surgery is uncommon, and confirming PSF absorption requires an extended amount of time. Nevertheless, this study is significant for analyzing the factors associated with PSF after SB surgery in RRD patients.

In conclusion, this study demonstrates that PSF may persist even after SB surgery in patients with chronic shallow RRD, and external SRF drainage can be an effective method to reduce the amount of SRF, shorten the duration of PSF, and induce faster visual improvement.

## Data Availability

The datasets used and/or analyzed during the current study available from the corresponding author on reasonable request.
